# Is There a Role for Music Intervention in Reducing Anxiety and Pain in Males During Outpatient Flexible Cystoscopy? A Randomized Controlled Study

**DOI:** 10.5152/tud.2025.25027

**Published:** 2025-06-24

**Authors:** Atsushi Wanifuchi, Toshiaki Tanaka, Tetsuya Shindo, Yuki Kyoda, Kohei Hashimoto, Naoya Masumori

**Affiliations:** 1 Department of Urology, Japanese Red Cross Kushiro Hospital, Kushiro, Japan; 2 Department of Urology, Sapporo Medical University School of Medicine, Sapporo, Japan

**Keywords:** Anxiety, cystoscopy, music therapy

## Abstract

**Objective:**

To prospectively verify whether music intervention reduces the anxiety and pain in male patients undergoing outpatient flexible cystoscopy in a randomized control trial.

**Methods:**

A total of 100 male patients undergoing outpatient flexible cystoscopy from May to November 2023 were randomly assigned to the music intervention group or control group. Spielberger’s State-Trait Anxiety Inventory (STAI) was used to assess the anxiety. The primary outcome was difference in the change of STAI-S level before and after the flexible cystoscopy between the groups. The secondary outcomes were pain and satisfaction measured by visual analogue scale (VAS). In addition, physiological change was assessed between the groups.

**Results:**

A total of 93 patients (48 in the music group and 45 in the control group) were included in the final analysis. Baseline characteristics were comparable between the groups. The median change of STAI-S in the music group was significantly greater than that in the control group (−5 vs −2, *P* = .03). The music group showed significantly lower pain and higher satisfaction on VAS after cystoscopy compared with the control group. By contrast, the change of physiological parameters was comparable between the groups.

**Conclusion:**

Music intervention may be useful to reduce both pain and anxiety associated with outpatient flexible cystoscopy in male patients.

**Main Points**

- Two-thirds of the cohort showed a high or very high pre-procedural anxiety score.
- The reduction of the State-Trait Anxiety Inventory score was greater in music intervention group than in the control group.
- The music intervention significantly decreased the pain and enhanced the satisfaction compared to the control group.

## Introduction

Diagnostic cystoscopy is one of the most commonly performed procedures in urological practice. The direct visualization of the lower urinary tract provides essential information for the diagnosis of underlying pathology in the bladder, prostate, and urethra. Therefore, cystoscopy is widely indicated for patients with asymptomatic hematuria, patients needing postoperative survey for intravesical recurrence of urothelial carcinoma, and those with lower urinary tract symptoms (LUTS). In 2023, more than 400 cystoscopies were performed at the hospital and the number is further increasing annually. However, cystoscopy is invasive and inherently associated with pain and discomfort.^1^

Although the advent of small-caliber flexible cystoscopy has greatly improved patients’ pain and discomfort compared with rigid cystoscopy, it is still troublesome for most patients.^1^ Some patients are hesitant about undergoing the examination owing to a negative impression. The Bladder Cancer Advocacy Network Patient Survey Network evaluated research prioritization for patients and found that reducing the pain associated with cystoscopy was a high priority for patients with non-muscle-invasive bladder cancer.^2^ In colonoscopy, the fear of pain and discomfort has been found to be one of the most determinant factors that discourage patients from undergoing colonoscopy.^3^ Also, in cystoscopy, the procedural pain and apprehension may make patients nervous and rigid, thereby making it difficult to complete the examination. Thus, pain alleviation has been of great interest for both patients and healthcare providers. In order to consistently provide adequate healthcare, it has been a pivotal issue to optimize the pain management strategy and improve adherence.

For better pain relief, several distraction methods have been explored such as 2% lidocaine instillation,^4^ music listening, and^5^ simultaneous visualization of the procedure.^6^ However, it is still unclear which intervention by itself or in combination optimally alleviates the patient burden. Listening to music is a safe non-pharmacological intervention in contrast to the pharmacological approach, which is ubiquitous and easily delivered across different clinical situations even in a resource-limited setting. Clinical, behavioral, and neuroimaging research have revealed that music intervention provides beneficial effects on decreasing pain and anxiety.^7^^,^^8^ In 3 comparative trials in patients undergoing flexible cystoscopy, two showed evidence supporting the use of music intervention for reducing the level of pain and anxiety,^9^^,^^10^ whereas 1 recent study reached the opposite conclusion.^11^ Additionally, in recent trials using both rigid and flexible cystoscopes beneficial effects of music intervention remained controversial.^12^^,^^13^ To the authors’ knowledge it remains to be determined whether music intervention should be introduced into standard of care in outpatient setting. The aim of this randomized controlled trial is to assess the effect of music intervention on alleviating anxiety and pain in male patients undergoing cystoscopy in an outpatient setting.

## Material and Methods

### Trial Overview:

This prospective single-institutional randomized controlled trial was conducted at Japanese Red Cross Kushiro Hospital from May to November 2023 in Japan. This study was approved by the Japanese Red Cross Kushiro Hospital (Approval number: 2022-17; Approval datr: March 3, 2023) and carried out in accordance with the provisions of declaration of Helsinki. This trial was registered with University Hospital Medical Information Network (UMIN000050766) clinical trials registry: Japan.

### Participants:

Patients were eligible for participation if they were male aged 18 or more who underwent the diagnostic cystoscopy. Patients were excluded if they need any support to communicate in Japanese or if they had anxiety disorder, anatomical urethral abnormalities, or history of allergy to lidocaine.

### Randomization:

An automatic online cloud-based randomization system was used to achieve control and balance between groups. Randomization were performed using minimization method with adjustment factor of age and prior experience of cystoscopy. After obtaining the written consent patients were registered online and were randomly assigned in a 1:1 ratio to either receiving cystoscopy plus music intervention (music group) or cystoscopy only (non-music group). Thereby physician was notified of allocation results via e-mail immediately after the registration.

### Sample size:

Sample size was calculated with effect size of 0.79 based on the prior study in male,^9^ alpha error of 0.05 and a power of 80% to be 86 patients large enough to yield a significant difference using G*Power 3.1.9.7. Allowing for attrition rate of 10%, a total of 100 patients (50 per group) were scheduled to enroll in the study.

### Intervention:

In music group, patients were offered to listen preferred music. Patients were able to choose slow music from a list matched on trial characteristics in different genre from classic (major key) or popular music. Upon entering the procedure room music was initiated on a compact disc player beside the examination table and the sound volume was set to preferred level of each individual. Music was continued until exiting the room. In non-music group patients received standard of care without music intervention.

### Cystoscopy Procedure:

All cystoscopy in this study was performed by a single urologist in the same fashion. Patients received an oral explanation of procedure overview, expected course, and comorbidity in advance by physicians. Patients were placed in a supine lithotomy position on UR-7300 examination table (Takara Belmont, Osaka, Japan). Urethral meatus was sterilized with 10% povidone-iodine. Immediately after 10 mL of 2% lidocaine gel at room temperature was instilled into the urethra, the scope was inserted into the urethra. The CYF-VHA flexible video cystoscope (tip: 12.9Fr, shaft: 16.2Fr) with VISERA ELITE platform^®^ (Olympus Medical Systems, Tokyo, Japan) was used in the study. Procedure time was recorded at the commencement of intubation of cystoscope till the time of extubation. Patients were instructed to take a slow and deep breath and relax while the scope was introduced through the external sphincter to bladder. Patients were able to communicate with attending nurse if they have any questions or need help. No analgesic or sedation was applied in the study. No prophylactic antibiotic was administered in the study.

## Measures:

### Demographic:

The following baseline characteristic data of patients were obtained: age, height, weight, medical history, prescription drugs, allergies, and prior cystoscopy experience. Before the procedure the International Prostate Symptom Score (IPSS) was used for the assessment of LUTS. All patients were required to fill out IPSS in the waiting room. The degree of severity of each symptom were rated on a 0-5 point scale and quality of life (QoL) was rated on a 0-7 point scale. Overall total score was classified into mild (0-7), moderate (8-19) and severe (20-35).

### Anxiety:

State-Trait Anxiety Inventory (STAI) was used to assess pre- and post-procedural anxiety level.^14^ Post-procedural anxiety level was assessed immediately after the procedure and before giving the explanation for the results. The STAI is a 40-item self-reported questionnaire for measuring anxiety level of individual, which contains 2 sets of 20-item on a 1-4 point Likert scale to separately assess the level of state anxiety (STAI-S) and trait anxiety (STAI-T). High score indicates the increased level of anxiety with each score ranging from 20 to 80. The Japanese version of STAI was used in the study. Delta STAI-S was calculated as indicator of reducing anxiety by subtracting pre- from post-STAI-S. In addition, pulse rate (PR), systolic blood pressure (SBP), and diastolic blood pressure (DBP) were also recorded before and after procedure as a physiological measure of stress.

### Pain and Satisfaction:

Visual analogue scale (VAS) was used for quantitative assessment of the pain and satisfaction. Paper-based VAS in the study was constituted as a straight horizontal 10-cm line with verbal anchor at both ends (0 mm = no pain/ 100 mm = maximal pain, 0 mm = not at all satisfied/ 100 mm = extremely satisfied). Patients were instructed to make a vertical mark on straight horizontal 10-cm VAS at the magnitude of their procedural experience after flexible cystoscopy. The pain and satisfaction level were scored by measuring in millimeters the distance from 0 to the marked site.

### Outcome:

The primary outcome was difference in Delta-STAI-S level before and after the flexible cystoscopy between the groups. The secondary outcomes were: (1) patient pain level during procedure by VAS; (2) patient satisfaction level by VAS; (3) change of SBP, DBP and HR as objective stress-related measure; (4) procedural time; and (5) occurrence of adverse event such as urinary tract infection between the groups.

### Statistical Analysis:

The continuous variables are expressed as median (interquartile range) and the categorical variables are presented as numerical. Difference between the groups were analyzed using Mann-Whitney U test for continuous variable and chi-square test for categorical variable. A two-sided *P* value <.05 was considered statistically significant. All statistical analysis was performed using EZR, freely avaible statistical software based on R (Y Kanda, Saitama Medical Center, Saitama, Japan).

## Results

### Patients

**Figure 1.**
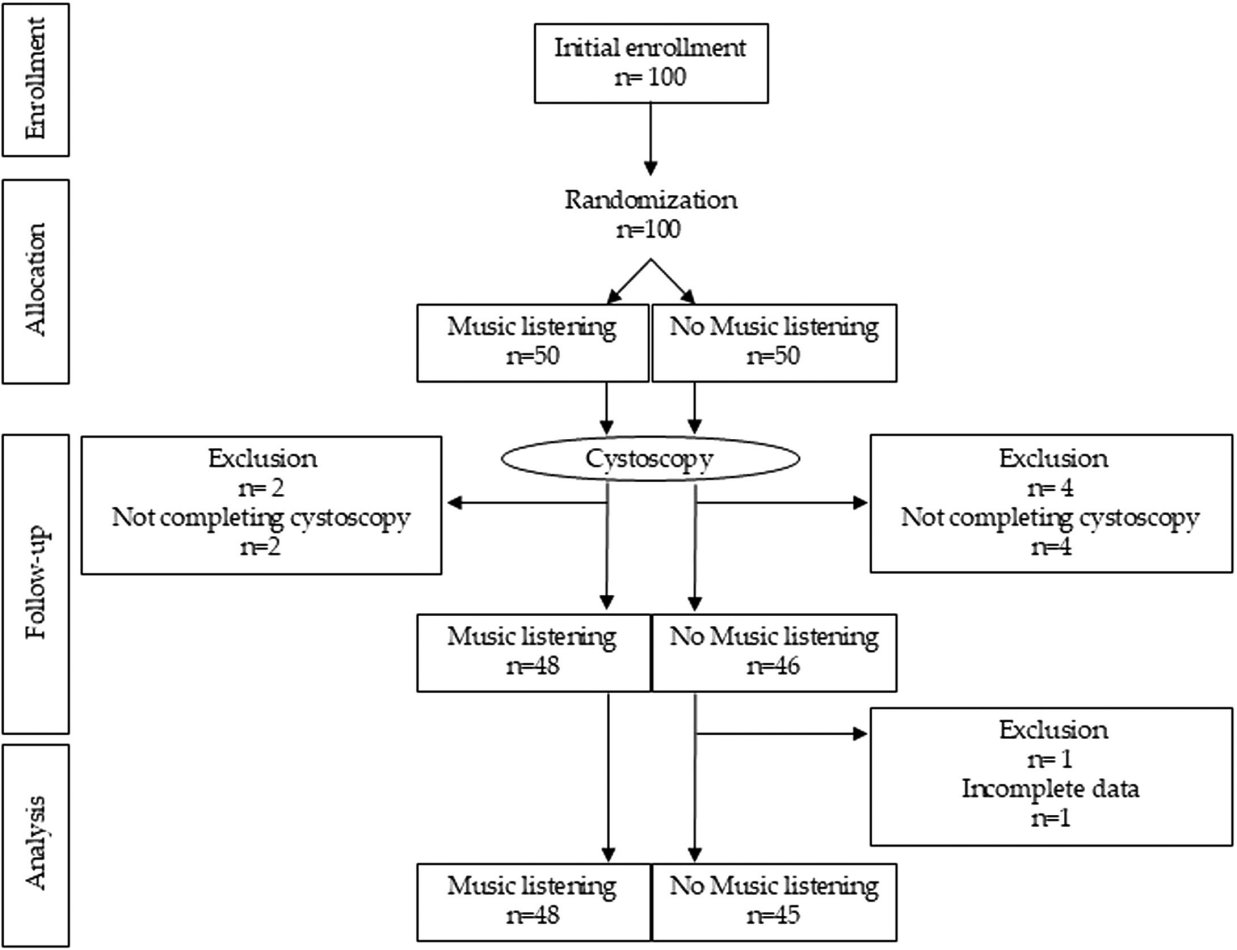
CONSORT participant flow diagram.

Between May and November 2023, a total of 100 patients were enrolled in the study. All patients were eligible and randomly assigned to receive either cystoscopy alone (non-music group, n = 50) or cystoscopy plus music intervention (music group, n = 50). Of these, the full cystoscopy procedure was completed in 94 patients, but not in 6 because of urethral stricture. One patient with incomplete data was excluded in the study. Thus, the remaining 93 patients were included in the final analysis. The Consolidated Standards of Reporting Trials (CONSORT) flow diagram and exclusion details of the study were shown in (Figure 1).

### Demographic Background

Baseline characteristics were comparable between the 2 groups regarding age, height, weight, BMI, analgesia use, and prior cystoscopy experience as shown in (Table 1). The most common indication for cystoscopy was hematuria in both groups. The median total IPSS score and QoL index at baseline were similar between the groups.

### Outcome:

The results of anxiety, pain, and satisfaction levels at pre- and post-procedural points are shown in (Table 2). A total of 62 patients (66.7%) exhibited high or very high pre-procedural anxiety scores, with 35 patients reporting high anxiety and 27 patients reporting very high anxiety on STAI-S. There was no significant difference in STAI-S, STAI-T, SBP, DBP, and PR at baseline between the groups. Decline in STAI-S was significantly greater in the music group than in the non-music group (−5 vs. −2, *P* = .03). The change in every physiological parameter between the groups did not reach statistical significance. In the music group, patients showed significantly lower pain and higher satisfaction on VAS after cystoscopy compared with the non-music group. No significant difference was observed in the procedure time between the groups (95.5 seconds vs. 88.0 seconds, *P* = .10). No infectious complications were observed in the entire cohort.

## Discussion

The present trial aimed to verify the efficacy of music intervention on reducing anxiety, leading to positive effects on pain, satisfaction, and physiological stress-related measures. As a result, a significant beneficial effect of music intervention on pain and anxiety perception was able to be detected, as well as satisfaction in males undergoing outpatient flexible cystoscopy compared with the control group. In contrast, physiological parameters did not show a significant difference between the groups. This is the first comparative study to clarify the efficacy of music intervention in male patients undergoing flexible cystoscopy in an outpatient setting. The results are consistent with prior trials of music intervention in male patients in an inpatient setting, indicating the analgesic and anxiolytic effects of utilizing music intervention in male patients undergoing flexible cystoscopy.^9^

Although the mechanism of the anxiety-reducing effect induced by music has not been fully elucidated, the involvement of the autonomic nerve system and limbic system is suggested.^15^ In the present study, a significant difference in STAI-S reduction was shown, but not in physiological parameters. This result is consistent with the meta-analysis showing that the impact of music intervention on physiological stress-related outcomes is relatively small compared with that on psychological stress-related outcomes, including patients undergoing various types of surgery.^16^ This predominance of psychological effects in response to music intervention was also observed in a gynecological setting.^17^

Pre-procedural STAI-S score was higher in the present study compared with prior 3 trials. Indeed, two-thirds of the patients showed high or very high anxiety scores. In the literature, young age and the degree of pre-provided information were associated with pre-procedural STAI-S in the urological procedure.^18^^,^^19^ Furthermore personality traits toward anxiety measured by STAI-T were reported to be associated with increased pre-procedural anxiety in central vein port placement and caesarean section.^20^^,^^21^ This is the first study of music intervention in Japanese patients undergoing cystoscopy. Baseline anxiety may be affected by nationality and ethnicity in addition to the environment and atmosphere of the clinic. Regardless of baseline characteristics, the results of this study suggest that music intervention is effective for reducing anxiety associated with outpatient cystoscopy.

Male patients were exclusively included, whereas 2 of 3 prior studies involved mixed-sex populations.^9^^-^^11^ A large-scale comparative study reported the mean pain level was significantly higher in males than that in females during diagnostic cystoscopy.^1^ Joo et al^13^ noted that gender differences are a potential confounding factor that should be controlled for in cystoscopy studies. Admittedly, the difference in pain perception between the sexes is largely responsible for the anatomical difference of the urethra. In addition, the reduction of the cold pressor-induced pain in response to preferred music was significantly greater in females than in males, suggesting that tolerability to pain and sensitivity to music intervention are different between the sexes.^22^ Besides sex differences, anthropometric factors can affect results in pain perception research. Weight is considerably reported to be associated with pain intensity.^23^ Given the nearly same diameter of the flexible cystoscope, physique may also be associated with pain sensation. In the current study, participants were randomized considering these factors. Therefore, the results of the study may demonstrate non-biased effects of music intervention on reducing pain associated with outpatient cystoscopy.

Music type or characteristic is an intuitively critical component that affects patient experience or outcome in a trial with music intervention. Some evidence suggests preferred music, unfamiliar music, sad music, tempo 60-80 beats per minute, music without lyrics are reported to be effective in decreasing pain in various settings.^18^^,^^24^^-^^26^ Recent meta-analyses showed that the analgesic effect does not depend on a specific type music in surgery.^24^^,^^25^ They found these results were attributed to scant descriptions of fundamental elements in music such as rhythm, tempo, melody, pitch, and harmony in prior trials. Indeed, Suda et al^27^ showed promising results that music listening reduces salivary cortisol level in stressful situation, and its effect was much greater in major music than in minor music with different pattern of brain activation using optical topography. Huang et al^7^ showed both happy music and neutral music ameliorated state anxiety with the different brain functional connectivity. Patient-selected music from list is also reported to be effective to sooth pain on meta-analysis.^25^ Psychological research showed self-selected music is effective in reducing stress as it gives a sense of control over the situation.^28^ However, Reynaud et al^17^ showed predetermined music was as effective as self-selected music. Basically, music is enjoyable but might negatively influence patient and be obstacle in communication.^29^ These indicate that the music selection should be determined based on patient preference or situation.

Although the mechanisms of music-induced analgesia are inconclusive, some possible explanation has been addressed in the literature. First, music intervention can simply distract patient attention from noxious stimuli. Its process was explained by cognitive coping strategy by way of attentional change that are accompanied by definite patterns of neurophysiological activity inhibiting the transmission of pain signal.^30^ Second, music-induced emotional reaction might explain pain reduction. It has been shown that pleasant music is associated with positive emotional valence, which results in decreased pain.^31^ However, in this literature negative emotional stimuli despite being both distractive did not affect pain experience. Similarly, Suda et al^27^ hypothesized music stimuli induces emotional response similar to a pleasant experience or happiness in upper temporal cortex and then results in stress reduction. These findings indicate music does not simply act as analgesia by distracting attention. Third, music listening might relieve pain through the descending pain analgesia pathway or the release of neurotransmitters. Some studies have demonstrated pleasant music is associated with increased mu-opiate receptor,^32^ increased dopamine release in striatal system.^33^ In the experimental study with functional magnetic resonance imaging pain stimuli plus music intervention showed significantly increased activity of the periaqueductal gray matter coupled with larger suppression in dorsal horn region compared with pain stimuli alone.^34^ On meta-analyses effect of music-induced analgesia was reported to be modest and equivalent to decrease a 10-23-mm VAS compared with control.^25^^,^^29^ This effect size was in accordance with this study. Conversely, these has been some promising results that music intervention might be more effective. For example, distraction group (music and/or toy) showed larger decrease in pain rating compared with ibuprofen in children with musculoskeletal trauma.^35^ Therefore, there may still be room for further optimization of music intervention, potentially as part of a multimodal approach alongside other supportive method.

This study has some limitations. First, this study addressed the small sample size and was performed by a single urologist at a single center, which limits the generalizability of the study. However, uniform procedure by a single operator appears to contribute to quality control as endoscopy is highly operator-dependent. Second, music intervention cannot be blinded to both patients and physician because of its nature, which increases the risk of bias in this trial.

In conclusion, it was demonstrated that music intervention significantly lightened the burden of both pain and anxiety in male patients undergoing flexible cystoscopy in an outpatient setting. Further evidence was added in different cultural population, supporting the therapeutic effect of music intervention during procedure. It is advisable music listening should be integrated in standard of care based upon patient preferences.

## Figures and Tables

**Table 1.** Baseline Characteristics

N	Music Group	Non-Music Group	*P*
	48	45	
Age (years)	74.5 (68.8-81)	72.0 (67-79)	.38
Height (cm)	166 (161-170)	163 (159-170)	.11
Weight (kg)	63 (58-69.3)	62 (56-70)	.57
BMI (kg/m^2^)	23.2 (21.0-25.1)	23.3 (21.3-26.4)	.53
Analgesia use (%)	18.7	22.2	.79
Indication for cystoscopy Hematuria Urothelial carcinoma follow-up LUTS	24 12 12	21 14 10	.86
Cystoscopy experience Naive Experienced	25 23	22 23	.83
IPSS			
Total score	9 (5-16)	10 (5-17)	.97
QoL index	4 (2.75-5)	3 (3-4)	.32

Data are shown in median (IQR). Chi-square test and Mann–Whitney *U-*test were used.

BMI, body mass index; IPSS, international prostate symptom score; IQR, interquartile range; LUTS, lower urinary tract symptom; QoL, quality of life.

*P < .05.

**Table 2.** Primary and Secondary Outcomes in Both Groups

N	Music Group	Non-Music Group	*P*
	48	45	
Time (sec)	95.5 (88.5-110.5)	88.0 (85-112)	.10
SBP (mmHg) Pre Post Delta (Post-Pre)	149 (132.2-158.5) 142.5 (128-155.2) −5 (−16.2 to 1.2)	143 (124-158) 140 (126-156) −3 (−10 to 6)	.20
DBP (mmHg) Pre Post Delta (Post-Pre)	83.5 (74-90.2) 82 (74-91.5) −1 (−6 to 6.2)	83 (76-93) 86 (79-92) 0 (−4 to 6)	.24
PR (bpm) Pre Post Delta (Post-Pre)	76 (69.5-86.2) 74 (66.7-83) −1 (−5 to 3)	79 (68-90) 73 (64-83) −2 (−6 to 1)	.09
STAI-S Pre Post Delta (Post-Pre)	45.5 (38.7-50.2) 41 (32.7-47.2) −5 (−10 to 0)	44 (38-49) 43 (35-49) −2 (−6 to 2)	.03
STAI-T	40.5 (35-45.7)	40 (35-45)	.86
VAS - Pain	19.5 (8.7-30)	31 (14-56)	.01
VAS - Satisfaction	79 (64.7-92)	70 (39-85)	.04

Data are shown in median (interquartile range).

DBP, diastolic blood pressure; PR, pulse rate; SBP, systolic blood pressure; STAI, State-Trait Anxiety Inventory; VAS, Visual Analogue Scale.

## Data Availability

The data that support finding of the results are available from corresponding author on a reasonable request.
